# Total knee arthroplasty after distal femoral osteotomy: a systematic review and current concepts

**DOI:** 10.1051/sicotj/2020033

**Published:** 2020-09-01

**Authors:** Francesco Luceri, Jacopo Tamini, Paolo Ferrua, Damiano Ricci, Cécile Batailler, Sébastien Lustig, Elvire Servien, Pietro Simone Randelli, Giuseppe Maria Peretti

**Affiliations:** 1 IRCCS Istituto Ortopedico Galeazzi Milan Italy; 2 FIFA Medical Center of Excellence, Orthopaedics Surgery and Sports Medicine Department, Croix Rousse Hospital, Civil Hospices of Lyon 103 Boulevard de la Croix Rousse 69004 Lyon France; 3 Humanitas Clinical and Research Center – IRCCS via Manzoni 56 20089 Rozzano, Milan Italy; 4 Azienda Socio Sanitaria Territoriale Centro Specialistico Ortopedico Traumatologico Gaetano Pini-CTO Piazza Cardinal Ferrari 1 20122 Milan Italy; 5 FIFA Medical Center of Excellence, Orthopaedics Surgery and Sports Medicine Department, Croix Rousse Hospital, Civil Hospices of Lyon 103 Boulevard de la Croix Rousse 69004 Lyon France; 6 LBMC UMR T 9406, Laboratory of Chock Mechanics and Biomechanics, Claude Bernard Lyon 1 University Lyon France; 7 LIBM, EA 7424, Interuniversity Laboratory of Biology of Mobility, Claude Bernard Lyon 1 University Lyon France; 8 U.O.C. 1a Divisione, Azienda Socio Sanitaria Territoriale Centro Specialistico Ortopedico Traumatologico Gaetano Pini-CTO, Piazza Cardinal Ferrari 1 20122 Milan Italy; 9 Laboratorio di Biomeccanica Applicata, Dipartimento di Scienze Biomediche per la Salute, University of Milan Via Mangiagalli 31 20133 Milan Italy; 10 Department of Biomedical Sciences for Health, University of Milan Milan Italy

**Keywords:** Distal femoral osteotomy, Lower limb alignment, Total knee arthroplasty

## Abstract

*Introduction*: Distal Femoral Osteotomy (DFO) is a common procedure for correcting lower limb valgus deformity and lateral compartment overload. Low 20-year survivorship rate was reported with a consequent need for total knee arthroplasty (TKA). This study aims to review literature and to analyse the influence of a previous distal femoral osteotomy on outcomes of patients undergoing TKA. *Methods*: A systematic literature review was performed in PubMed/Medline and Embase in May 2020. Papers were selected based on the following criteria: patient with a previous distal femoral osteotomy; total knee replacement; Pre- and Postoperative outcomes; surgical outcomes: clinical scores, range of motion, radiographic evaluation and revisions for any cause; case series, retrospective studies, observational studies, open-label studies, randomized clinical trials; systematic reviews and meta-analyses were included to extract primitive studies. *Results*: 306 articles were found, of which five papers were considered eligible for this review. In every study included, postoperative clinical outcomes (Knee Society Score or Hospital for Special Surgery score) statistically improved from the preoperative. Complications were not uncommon; implant survivorship at the available follow-up seems to be similar to primary TKA, although being too short to draw any conclusions. *Conclusions*: Limited and highly heterogeneous evidence is currently available on the influence of DFO on outcomes after TKA. Knee replacement improves clinical middle-term outcomes in patients with previous distal femoral osteotomy. In this complex surgery, the use of technical tips and tricks could help surgeons to obtain an accurate knee balancing and better long-term results.

## Introduction

Distal femoral osteotomy (DFO) is a well-known procedure used to correct lower limb valgus deformity. This surgery aims to reduce lateral compartment overload and to prevent knee osteoarthritis (OA) progression [1].

DFOs can be performed with a medial closing wedge (CWDFO) or a lateral opening wedge (OWDFO) technique. OWDFO is technically easier but requires bone or synthetic grafting to fill the osteotomy gap and has a 5% risk of delayed union [2].

For these reasons medial closing wedge osteotomy is usually preferred for larger corrections and in patients with high risk of non-union, whereas lateral opening wedge DFO can be chosen for less than 10° corrections [3].

Limited and highly heterogeneous body of literature was found to exist for both opening- and closing-wedge DFO.

A mean survival rate of 58% at 15 years and 21.5% at 20 years follow-up was reported for closing-wedge DFOs. Likewise, opening-wedge survival rate of 84%–100% was reported at 8-year follow-up. Hardware-related issues are the most prevalent complications of these surgical procedures [4, 5].

Studies showed that the 20-year survivorship of a DFO is about 22%, with a mean time of conversion to total knee arthroplasty (TKA) of 10–15 years; [3] this means that in the future, many surgeons will implant TKA in patients with previous DFO. Despite the excellent results of TKA, it may be difficult to recommend an arthroplasty as the primary intervention for these patients particularly in those intending to return to sports [6].

While different reviews have been published about performing a knee replacement after a failed HTO, fewer studies tried to analyse technical difficulties of TKA after DFO and outcomes of knee replacement in this particular group of patients.

This review wants to be the first study to group all papers published about this topic in order to update knowledge and to show all possible risks and complications of this procedure.

The aim of this systematic review was to analyse the influence of DFO on outcomes of patients undergoing TKA for knee OA.

## Materials and methods

A systematic literature review was performed following the PRISMA statement for transparent reporting of systematic reviews and meta-analyses [7].

The following participants, interventions, comparisons, outcomes, and study (PICOS) design were used: patient with a previous distal femoral osteotomy (P); total knee replacement (I); Pre- and Postoperative outcomes (C); outcomes of surgery: clinical scores, range of motion, radiographic evaluation and revisions for any cause (O); case series, retrospective studies, observational studies, open-label studies and randomized clinical trials. Systematic reviews and meta-analyses were included to extract primitive studies (S).

Database search included Medline/PubMed, Embase in May 2020. PubMed database was searched for the terms “femoral osteotomy” [All Fields] AND (“knee replacement” [All Fields] OR “knee arthroplasty” [All Fields]). Embase database was searched for the terms (“knee arthroplasty”/exp OR “knee arthroplasty” OR “knee replacement”/exp OR “knee replacement”) AND (“femoral osteotomy”/exp OR “femoral osteotomy”).

Exclusion criteria were: Papers in a language different than English, case reports, commentaries, letters to the editor, biomechanical reports and studies in which computer-assisted knee replacement was performed.

Results were firstly checked by title and abstract in order to exclude studies not related to this topic, and for those still suitable the full text was collected to establish the coherence with the purposes PICOS of this review. Two different independent Authors (FL and JT) performed these steps and results were then matched, resulting as comparable.

## Results

**Figure 1:**
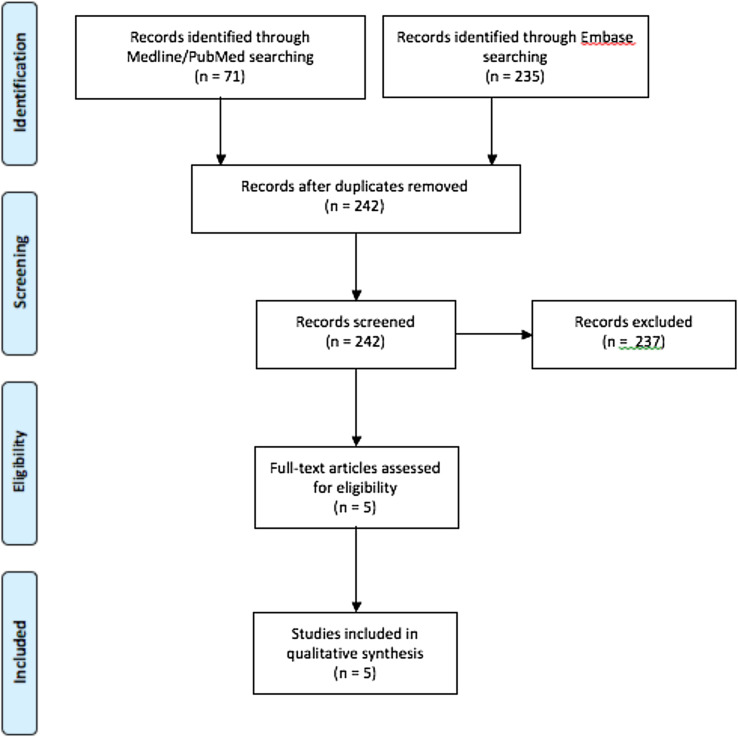
Study flow-chart.

Three-hundred six articles were found using the described research strategies (71 articles in PubMed/Medline and 235 in Embase). Duplicates were recognized and deleted using the Mendeley program, reaching the number of 242 articles. After checking titles, abstracts and full texts, 237 articles were then excluded reaching a final number of 5 papers [8–12] eligible for this review (Figure 1).

Five studies (4 retrospective observational studies and 1 retrospective case–control study) that considered total knee arthroplasty after distal femoral osteotomy were included in this review (Table 1). Bias assessment was not possible due to the design of the selected studies.

**Table 1:** Comparison of eligible studies.

Author	Study design	Sample size	DFO	Osteotomy correction	Surgical approach	Outcomes	Follow-up
Cameron et al. [10]	Retrospective observational	8 patients (F: 5; M: 3); mean age; 54.8 y.o. (40; 66); BMI: N/R	7 Medial CWDFO – Subvastus approach 1 Lateral OWDFO + tibial osteotomy Average interval between DFO and TKA: 4 years	Mean: 12°	7 Medial parapatellar approach 1 Lateral subvastus approach – Tricon knee	HSS in 5 excellent, in 3 good Post-TKA ROM: > 100°; Mean HKA: valgus 7° (5°;9°) Complication: 1 revision after 6 years (polyhetilene wear), 1 MUA for stiffness, 1 reflex sympathetic dystrophy	4 years (range 2–9)
Chalmers et al. [11]	Retrospective observational	29 patients (F: 17; M: 12), 31 knees; mean age: 51 y.o. (22; 76); BMI: 32	17 Lateral and 14 Medial DFO; 25 varus- and 6 valgus-producing Average interval between DFO and TKA: 10 years (2; 20)	N/R	N/R – 24 PS implants; 4 Varus-Valgus constrained; 3 CR implants	PreOp KSS: 50 (32 to 68) PostOp KSS: 93 (76; 100) Post-TKA Flexion: 117°; 88% ten-year implant survivorship. Complications: 3 knee stiffness; 1 patella fracture, 1 aseptic loosening, 2 instabilities, radiolucency in 3 TKAs	10 years (range 2–16)
Gaillard et al. [9]	Retrospective case-control	14 patients (F: 2; M: 12); median age: 66.6 y.o (43-89); BMI: 27.2	14 Lateral OWDFO – Lateral approach Average interval between DFO and TKA: 18.8 years (6; 63)	Surgical goal: residual varus 0-3°	Medial (11) or Lateral (3) parapatellar approach – 14 PS implants	PreOp KSS knee: 46.3 PreOp KSS function: 59.3 PostOp KSS knee: 91.7 PostOp KSS function: 70.6 Post-TKA Flexion: 116° mFTA 177° Complications: -IntraOp: lateral laxity (2), medial laxity (2), patella tendon injuries (4), need for screw support for the tibial component (2) -PostOp: 1 skin necrosis	Median: 41.8 months (range 12–102)
Kosashvili et al. [8]	Retrospective observational	21 patients (F: 17; M: 4), 22 knees; mean age: 56 y.o. (42; 71); BMI: N/R	N/R − Subvastus approach + Fresh chondral allograft transfer (7 knees) Average interval between DFO and TKA: 13 years (3; 23)	Varus-producing	N/R – Stemmed components in 5 knees (2 femoral and 3 tibial components) NexGen posterior-stabilized implant (Zimmer Inc, Warsaw, Ind)	PreOp KSS: 49 (10;75) PostOp KSS: 91 (67; 100) Post-TKA (unrevised only) ROM: 114° (90°;130°) PostOp Install-Salvati Ratio: 1.00 (0.68;1.42) Complications: 4 patella baja, 2/7 with previous fresh osteochondral allograft revised for component loosening: 1 polyethylene wear (8 years) + instability + osteolysis around tibial and femoral component, 1 aseptic loosening of tibial component (at 12 years), radiolucency in all the TKAs	5 years (range 2–14)
Nelson et al. [12]	Retrospective observational	9 patients (F: 7; M: 2), 11 knees; mean age: 44 y.o. (15; 70); BMI: N/R	4 medial, 5 lateral, 2 N/R Average interval between DFO and TKA: 14 years (2; 32)	Varus-producing - 6 varus, 2 neutral, 1 valgus knees	9 medial, 2 lateral arthrotomy - 1 Insall-Burstein (Zimmer), 2 Insall-Burstein II prostheses (Zimmer), and 2 PFC PS (DePuy); 5 CCK; 1 rotating-hinge prosthesis (FINN; Biomet)	PreOp KSS: 35 (13; 58) PostOp KSS: 84 (71; 93) Post-TKA ROM: 105.9° (90°; 125°) Radiographic alignment: 3.6° PreOp valgus (7° varus; 18° valgus); 3.3° of PostOp valgus (1°; 6° valgus) Complications: 1 intraoperative femur fracture, 1 patellar clunk (4 years); 1 death not related to surgery, radiolucency in all the TKAs	5.1 years (range 2.5-18)

F female, M male, y.o. years old, N/R not reported, DFO Distal Femur Osteotomy, CWDFO Closing Wedge Distal Femur Osteotomy, OWDFO Opening Wedge Distal Femur Osteotomy, TKA Total Knee Arthroplasty, HSS Hospital for Special Surgery rating, ROM Range Of Motion, KSS Knee Society Score, MUA Manipulation Under Anaesthesia.

The aim of Cameron et al. [10] was to investigate the outcomes of TKA after DFO; authors concluded that, in most instances, previous correctly done supracondylar osteotomy does not reduce the success rate of TKA.

The study by Chalmers et al. [11] wanted to report long-term results and survivorship of cemented TKA after DFO; they observed reliable improvement in clinical outcomes with primary implants and selective utilization of femoral stems; 13% of TKAs required a varus-valgus constrained implant, and 6% revision rate for implant instability was reported.

Gaillard et al. [9] aimed to compare clinical and radiological outcomes of TKAs performed after varus DFO with a control group of TKAs in patients not previously treated with osteotomy. Authors concluded that TKA after DFO delivers excellent results, comparable to the control group.

The purpose of the study of Kosashvili et al. [8] was to analyse the outcomes of TKA performed after successful varus DFO and to assess the necessity of constrained prostheses and stemmed components in these patients. They concluded that an appropriate ligamentous balancing is mandatory to provide satisfactory stability in TKA after varus DFO, without the need for stemmed or constrained components in the majority of the cases.

The goal of the study by Nelson et al. [12] was to evaluate the intermediate-term results of TKA in patients with a previous DFO and authors conclude that this procedure decreases pain and improves knee function, but results are inferior when compared to primary arthroplasties.

## Discussion

The main finding of this review is a severe lack of scientific literature about the effect of DFO on clinical outcomes of TKA surgery. No randomized or non-randomized controlled trials analysing this aspect were eligible for this review. Four retrospective observational studies and one retrospective case–control study were identified. Non-randomized observational studies in general provide a low quality of evidence. Finally, the risk of bias due to several study limitations further reduces the quality of evidence.

Knee replacement seems to improve clinical middle-term outcomes even in patients who underwent previous DFO.

### Sample size

All the included studies [8–12] analysed a low number of patients. Future studies will surely have a larger sample size.

In four out of five studies there were more females than males. This may be because valgus knee is a typical female anatomical feature.

Age seems to be relatively low in all groups with respect to patients undergoing primary knee replacement, and this could be explained with the fact that some of these patients had a symptomatic constitutional knee deformity.

### Previous osteotomy

Most of the included patients underwent a lateral DFO rather than medial one. In four studies [8, 9, 11, 12] the average interval time between DFO and TKA was more than 10 years. In the other paper [10], the mean time was 4 years. The more dated surgical technique and available hardware analysed in that study could explain the different outcomes.

### Hardware removal

In four of the five studies analysed [9–12], staged hardware removal was performed before TKA in 20.8% of patients; 59.7% of hardware were removed during TKA and in 19.4% of cases they had been left in-situ. One study did not mention this specific aspect of the surgery [8].

While simultaneous removal may be preferable to avoid multiple surgeries, staged surgeries may be the best option in suspicion of hardware-related pain or subclinical local infection.

### Knee replacement

PS implants were mostly used for knee replacement, sometimes adding stems.

In one study [9] the use of screws for supporting tibial component was sometimes requested and in another [8] fresh osteochondral allografts were requested for 7 patients.

These are different options that may be used in cases of inadequate bone-stock.

### Complications

Intraoperative complications were described for 11 patients: lateral laxity (2 cases), medial laxity (2 cases), patella tendon injuries (4 cases), need for screw support for the tibial component (2 cases), and 1 intraoperative femoral fracture [11].

Postoperative complications included: 1 polyethylene wear, 6 stiffness (2 concomitant patella baja), 1 reflex sympathetic dystrophy, 1 patellar fracture, 3 aseptic loosening, 2 instabilities, 1 skin necrosis and 1 patellar clunk [11].

The overall intra-operative complication rate was 12.8% while the post-operative one was 18.6%.

### Follow-up

The average follow-up after knee replacement was between 3 and 5 years [8–10, 12], while only one study reported a follow-up of 10 years [11].

Knee replacement in patients with a previous DFO needs to take into account different factors such as prior skin incisions, joint exposure, previous hardware, bone deformities, ligamentous balance and component placement.

DFO is a technically demanding surgery and subject to several postoperative complications including stiffness and patellofemoral arthritis [13], nevertheless femoro-tibial arthritic progression, however, is very common.

TKA after DFO has several technical issues. Preoperative stiffness may limit exposure during the surgical procedure, pre-existing scar tissue may be extensive and carry a risk skin necrosis, and ligament balancing, particularly in flexion, may be challenging. Constrained prosthesis may be used to avoid residual knee instability.

Rotational abnormalities of the femur are very common undiagnosed complication after DFO; It is a induced mistake in rotation by the osteotomy [14]. This is usually not detected except with CT scan [15]. For these reasons, an accurate preoperative CT study, after the metal implant removal, could be mandatory to obtain a complete preoperative TKA planning.

About surgical approach, care must be taken to avoid skin necrosis; if the previous incision is inadequate for knee replacement, a standard midline approach can be utilized maintaining an adequate skin bridge.

In case of difficult bone exposition, quadriceps snip or tibial tubercle osteotomy may be used to avoid excessive tensioning of the extensor apparatus.

Previous hardware may be removed before or during TKR, but may also be left in situ in asymptomatic patients if there is no implant impingement.

When using an intramedullary femoral alignment, the femoral entry point should be more lateral than usual to ensure that the intramedullary rod is aligned with the femoral diaphysis. Preserving the degree of femoral varus seems to allow simple and reproducible ligament balancing during TKA surgery [9]. The use of intramedullary femoral guide increased the tendency to place the femoral component in relative varus angulation [12].

None of the analysed studies focused on the effect of DFO on the sagittal knee alignment or on the patellar height [16].

Although the aim of our review was to find evidence on a very specific topic, results are consistent with a lack of evidence. These findings do not necessarily mean a lack of positive correlation between DFOs and TKAs.

Surgeries can be technically more difficult than a primary knee replacement, both for bone-stock or bone-quality insufficiency and for possible severe intra-articular deformities due to pre-existing conditions and previous surgeries.

Outcomes and long-term survivorship cannot be compared with those of primary knee arthroplasties because of inadequate follow-up of studies involving this group of patients.

## Conclusion

The results of this systematic review demonstrate that limited and highly heterogeneous evidences are currently available on the influence of DFO on outcomes after TKA surgery.

Knee replacement seems to improve clinical middle-term outcomes in the patients. This is a complex surgery and the use of specific technical tips and tricks could help the surgeon to obtain a more accurate knee balancing and perhaps better long-term results.

Future prospective studies and comparative trials should be designed to specifically address the impact of DFO in knee replacement surgery, evaluating long-term implant survival rate and joint function.
